# The changing landscape of rabies epidemiology and control

**DOI:** 10.4102/ojvr.v81i2.731

**Published:** 2014-04-23

**Authors:** Sarah Cleaveland, Hawthorne Beyer, Katie Hampson, Daniel Haydon, Felix Lankester, Tiziana Lembo, Francois-Xavier Meslin, Michelle Morters, Zacharia Mtema, Maganga Sambo, Sunny Townsend

**Affiliations:** 1 Boyd Orr Centre for Population and Ecosystem Health, Institute of Biodiversity, Animal Health and Comparative Medicine, College of Medical, Veterinary and Life Sciences, University of Glasgow, Scotland; 2 ARC Centre of Excellence for Environmental Decisions, Centre for Biodiversity and Conservation Science, University of Queensland, Australia; 3 Ifakara Health Institute, Ifakara, Tanzania; 4 Paul G. Allen School of Global Animal Health, Washington State University, United States; 5 Lincoln Park Zoo, Chicago, United States; 6 World Health Organization, Geneva, Switzerland; 7 Department of Veterinary Medicine, University of Cambridge, United Kingdom

## Abstract

Over the past 20 years, major progress has been made in our understanding of critical aspects of rabies epidemiology and control. This paper presents results of recent research, highlighting methodological advances that have been applied to burden of disease studies, rabies epidemiological modelling and rabies surveillance. These results contribute new insights and understanding with regard to the epidemiology of rabies and help to counteract misperceptions that currently hamper rabies control efforts in Africa. The conclusion of these analyses is that the elimination of canine rabies in Africa is feasible, even in wildlife-rich areas, through mass vaccination of domestic dogs and without the need for indiscriminate culling to reduce dog population density. Furthermore, the research provides valuable practical insights that support the operational planning and design of dog vaccination campaigns and rabies surveillance measures.

## Introduction

Over the past 20 years, much progress has been made in understanding the epidemiology of rabies in Africa, supporting the view that canine rabies elimination is both feasible and cost-effective through mass vaccination of domestic dogs (Lembo *et al*. 2010; Rupprecht *et al*. 2008; World Health Organization [WHO] 2004; WHO 2013). This research has resulted in a groundswell of momentum amongst rabies scientists and international human and animal health agencies to drive forward ambitious plans for canine rabies elimination. However, despite huge progress in some parts of the world, most notably Latin America where canine rabies elimination is targeted for 2015 (WHO 2012), substantial challenges remain in Africa where few national programmes are in existence.

**Figure 1:**
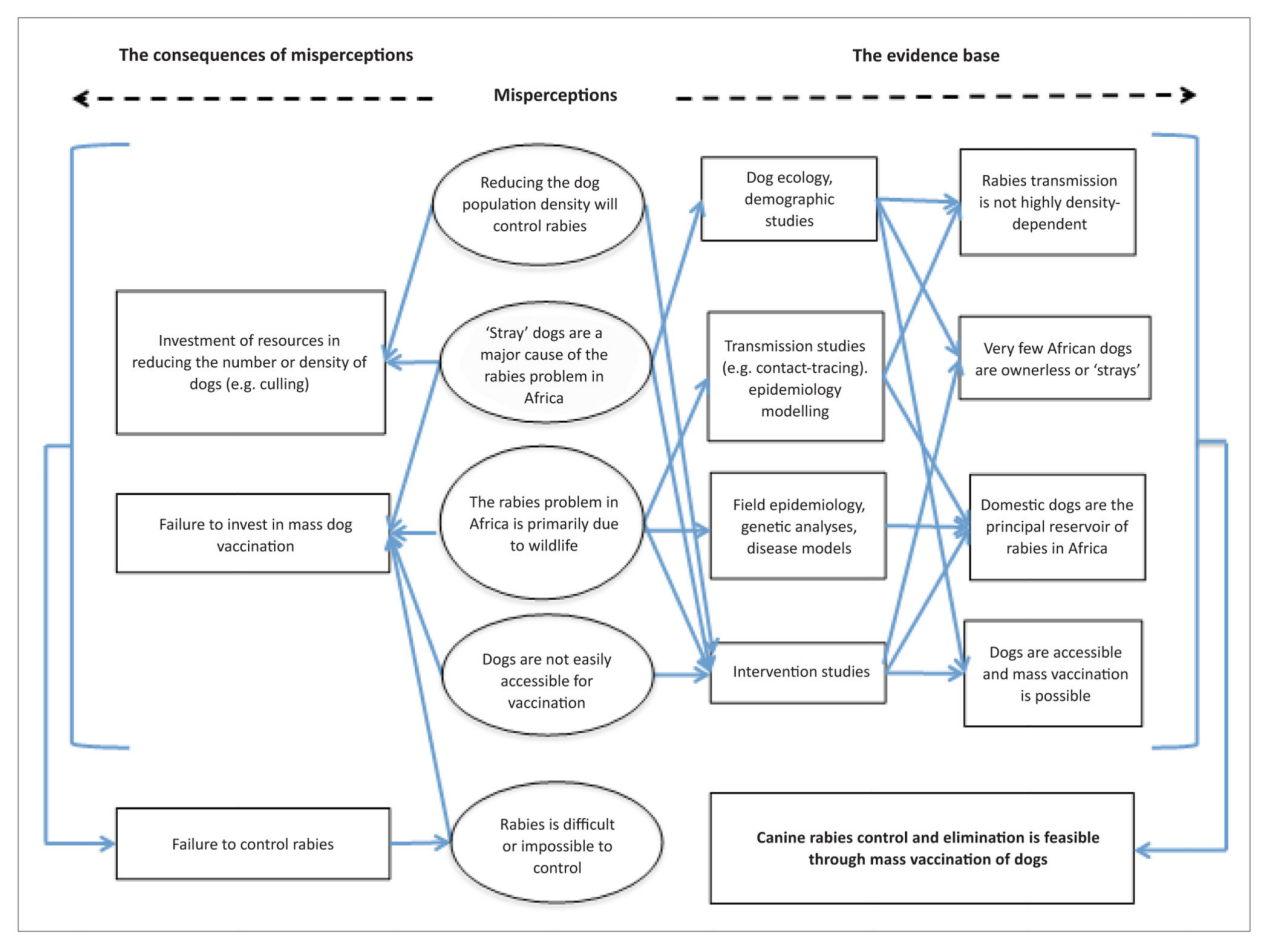
Scheme showing how misperceptions about dog ecology and rabies epidemiology have negative consequences for rabies control; and identifying the types of research studies that can generate the evidence-base needed for effective control.

An enduring problem relates to several misperceptions that currently hamper rabies control efforts in Africa (Lembo *et al*. 2010) (Figure 1). For example, there is a perception that rabies is relatively insignificant as a disease of public health concern; that rabies is a problem of ‘stray’ dogs that are not accessible for parenteral vaccination; that rabies can only be controlled through culling or reduction in the dog population density; and that wildlife play a major role in sustaining rabies cycles in Africa. These misperceptions drive a cycle of neglect, where the implementation of ineffective control measures results in demotivation of policy-makers and veterinary field staff and fuels the erroneous impression that rabies control is futile.

Here we review the most recent evidence from rabies epidemiology studies that address these misperceptions, highlighting how new approaches and analytical techniques are being applied and illustrating how the results are constantly reinforcing the evidence base as to the feasibility of canine rabies elimination in Africa.

## Rabies burden of disease studies

In common with many ‘neglected’ diseases, a principal factor contributing to the low prioritisation of rabies control has been the lack of information about the burden and impact of rabies, particularly in low-income countries that are most affected by the disease. The development of a probability-tree model for estimating human rabies deaths from the incidence of suspect rabid animal bite injuries and human post-exposure prophylaxis (PEP) (Cleaveland *et al*. 2002) has paved the way for several estimates of human rabies incidence in Africa and Asia (Coleman, Fèvre & Cleaveland 2004; Fèvre *et al*. 2005; Hossain *et al*. 2012; Knobel *et al*. 2005; Ly *et al.* 2009; Tenzin *et al*. 2011). These studies have indicated that rabies incidence in Africa, estimated to be ~24000 deaths per year (Knobel *et al*. 2005), is at least 100 times higher than officially reported.

The most recent estimates of rabies burden incorporate more detailed country-level data, including updated information on human PEP use. These analyses, part of the global burden of disease studies coordinated by the Partners for Rabies Prevention, have also included consideration of economic losses as a result of premature deaths. Overall, using the probability model, global estimates of human rabies deaths are similar to previous studies, with an estimated 61 000 human rabies deaths per year occurring globally (95% CI, 52 200–70 700) and 23 800 in Africa (95% CI, 21 000–28 000) (WHO 2013). However, the apparent consistency in estimates masks substantial local variation, most notably the decline in human rabies deaths in several countries in Asia because of increased availability of rabies PEP and, in some areas, dog rabies control. Whilst any decline in human rabies deaths is to be celebrated, this progress has come at a high cost, with PEP costs in Asia estimated at around US$1.5 billion. These contribute substantially to the estimated global annual cost of rabies of US$6bn (95% CI, 4.6–7.3), which also includes productivity losses of US$2bn as a result of premature deaths (WHO 2013).

Empirical data to both parameterise and validate estimates of human rabies deaths have been generated from community surveys (Hossain *et al.* 2012), large-scale verbal autopsy surveys (Suraweera *et al*. 2012) and active surveillance and contact tracing (Hampson *et al*. 2008). Nonetheless, it is clear that these data are still very scarce and that any figures based on models that incorporate incomplete and imprecise data are likely to have a high degree of uncertainty.

Despite these uncertainties, clinical studies in Malawi have demonstrated that rabies is a more common cause of childhood encephalitis than recognised previously and can be misdiagnosed easily as malaria, even by experienced clinicians (Mallewa *et al*. 2007). This may seem surprising, given that rabies exhibits several distinct clinical features in humans, such as aerophobia and hydrophobia, but serves to highlight the importance of improving awareness amongst clinicians and submitting samples for laboratory diagnosis to ensure that the rabies disease burden is not masked by high levels of misdiagnosis, particularly in malaria-endemic areas of Africa.

**Figure 2:**
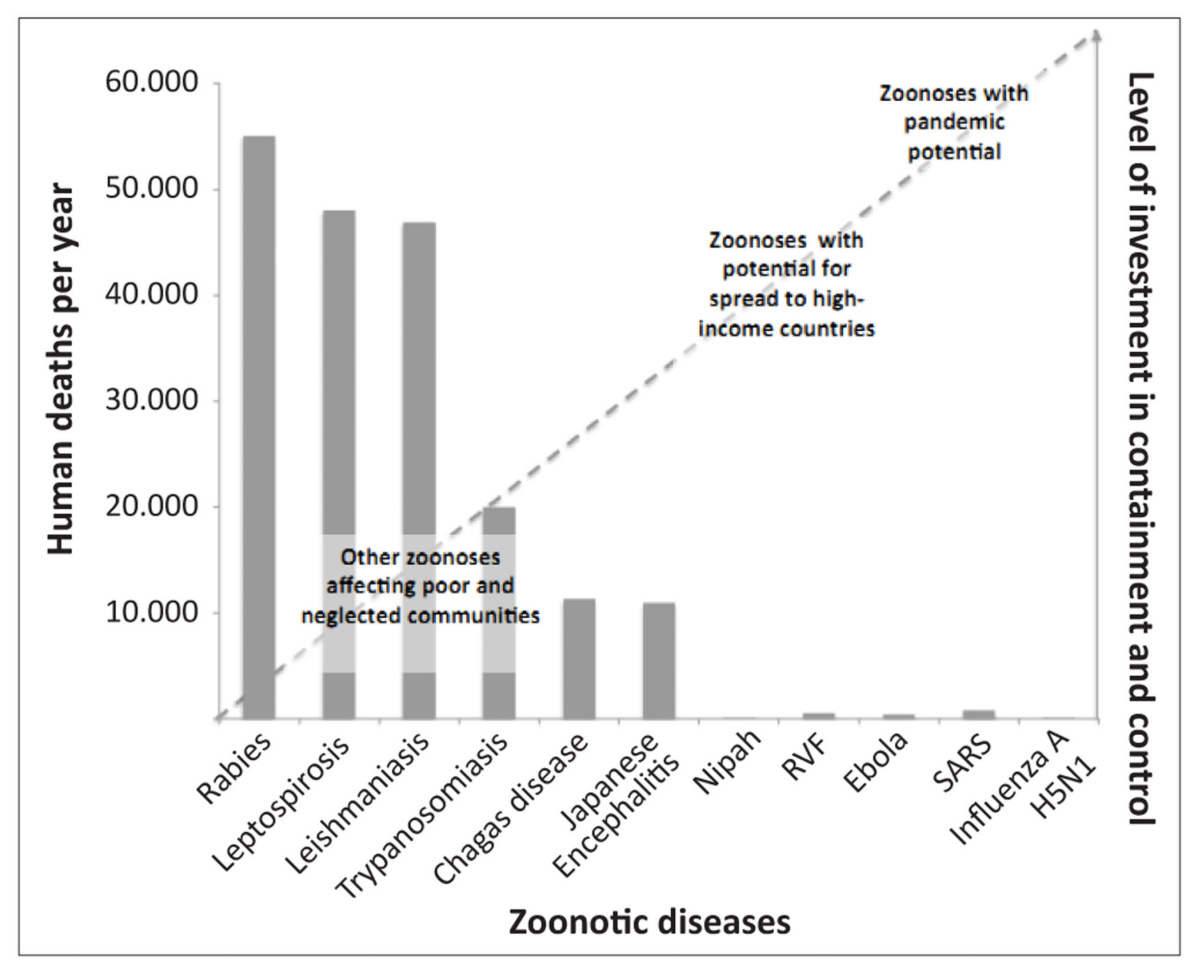
Figure showing the estimated annual number of human deaths from major zoonotic diseases in relation to the level of investment spent in containment and control. *Source*: adapted from Lembo *et al*. (2010) RVF, Rift Valley Fever; SARS, Severe Acute Respiratory Syndrome.

Although rabies does not have the pandemic potential that characterises the emerging zoonoses of greatest concern to high-income countries (Figure 2), its burden and impact cannot be considered negligible. The physical, psychological and economic consequences of rabies are borne by the poorest communities in the world and the continued suffering caused by the disease is entirely preventable and remains a conspicuous failure of the veterinary and medical professions to deliver on existing solutions.

### Epidemiological modelling

The advent of powerful analytical and modelling tools now allows valuable insights regarding the epidemiology and control of rabies to be generated from a wide variety of epidemiological, genetic and geographic data. Patch occupancy models, developed from metapopulation theory to explore persistence of species in fragmented habitats (Hanski & Ovaskainen 2000), have recently been applied to the study of rabies persistence in structured populations in the Serengeti ecosystem. In this study, hospital-derived animal-bite injury cases were used as a measure of disease occurrence in villages (‘patches’), exploiting an easily accessible source of data to parameterise spatially-explicit models of disease on a regional scale (Beyer *et al*. 2011). Bite-injury case records typify the type of coarse field data that is often the only information available to epidemiologists in Africa. These data contain many uncertainties that have previously limited their utility for epidemiological inference. However, the use of state-space models provides a powerful framework for modelling the effects of uncertainty and have allowed us to exploit accessible but ‘noisy’ data in order to generate new insights into regional-scale transmission dynamics. For example, the Serengeti model highlighted the importance of spatial structure, with village-to-village transmission of dog rabies being more important in driving regional-scale dynamics than transmission from wildlife-protected areas; and being consistent with results of earlier studies that identified dogs, not wildlife, as being rabies reservoirs in the Serengeti (Lembo *et al*. 2008). These models also have potential for the design of spatially-structured vaccination campaigns by identifying communities that contribute most to the persistence of rabies; they could, therefore, be prioritised for vaccination in situations where resources are limited (Beyer *et al*. 2012).

Whilst much effort has focused on understanding the epidemiology and control of rabies in endemic settings, attention is now turning toward prospects for the elimination of canine rabies. The feasibility of canine rabies elimination has been supported by a strong body of evidence from epidemiological field studies, phylogenetic analyses and epidemiological modelling (summarised in Lembo *et al*. 2010). A key finding has been the low value of *R_0_*, which consistently falls between 1.0 and 2.0 for canine rabies in dog populations across the world, despite wide variation in dog densities and demographic characteristics in urban and rural communities in different countries (Hampson *et al*. 2009). The lack of density-dependence in rabies transmission was evidenced again with an *R_0_* estimate of 1.2 for the 2008–2010 rabies outbreak in Bali, Indonesia in communities with very high dog densities (Townsend *et al*. 2013).

These findings have two major implications for rabies control and elimination: firstly, that elimination of canine rabies through vaccination of ~70% dogs is epidemiologically feasible in most settings, including high-density populations; and secondly, that tackling rabies through dog density reduction (e.g. mass culling) is likely to be ineffective (Morters *et al*. 2013). These conclusions are also supported by previous experience. For example, despite substantial reductions in the dog population of Flores, Indonesia as part of a culling program to control a rabies outbreak in 1996, the disease remains endemic (Windiyaningsih *et al*. 2004). Similarly, culling failed to control canine rabies in Korea, Israel, and Bali, whereas subsequent mass dog vaccination programmes have resulted in control of the disease (Knobel *et al*. 2013). In addition to having no demonstrable beneficial impact on the control of rabies, indiscriminate culling of dogs also has substantial ethical and welfare implications and no evidence exists to support its use in rabies control strategies. However, misperceptions remain widespread (presumably because conclusions from research studies run counter to many intuitive assumptions) and culling is still considered a feasible policy option by many veterinary services in Africa. Engaging with policy-makers therefore remains a high priority to ensure uptake of research findings and to instill confidence as to the feasibility of rabies control and elimination through mass dog vaccination.

Epidemiological models also provide critical support for the development and design of elimination strategies in terms of understanding elimination dynamics; designing the most cost-effective strategies for disease control and surveillance at national, regional and global levels; and providing guidance for policymakers about expected times to elimination. A spatially-explicit stochastic simulation model of rabies in Bali, Indonesia, for example, has indicated that time to elimination of rabies is critically dependent on high levels of contiguous vaccination coverage and that even small pockets of low coverage (e.g. involving < 0.5% of the dog population) can cause a significant delay in progress (Townsend *et al*. 2013). In contrast, the probability of rabies elimination is only slightly affected by the timing of campaigns (e.g. intensive synchronised versus more prolonged dog vaccination campaigns), so campaigns can be tailored to optimise logistic efficiency and resource availability in order to achieve the high vaccination coverage required.

### Surveillance approaches

Surveillance is a critical element of the control and elimination of infectious diseases. As control programmes progress toward elimination, surveillance efforts need to be intensified in order to detect new incursions and to declare freedom from disease with confidence. Surveillance remains arguably the weakest element of many national and regional control and elimination strategies, particularly in Africa and Asia, with well-recognised difficulties associated with detection and confirmation of both animal and human cases. A further problem relates to the potentially long incubation period of rabies (Fekadu 1993; Hampson *et al*. 2009), making it difficult to determine whether rabies has truly been eliminated during periods with no detected cases.

Several questions therefore remain as to the optimum control strategies that should be adopted in the face of low case-detection probabilities, as well as regarding the duration and level of surveillance efforts that are needed in order to provide confidence that rabies has truly been eliminated. Although definitions for freedom from canine rabies have been proposed (WHO 2013), current international guidelines lack quantitative definitions about the level of surveillance that would be required by an effective surveillance system for the purpose of declaring freedom from rabies (OIE 2011). Recent modelling work provides useful insights, for example, in the use of outbreak simulation techniques to explore the effectiveness of control and elimination strategies. In relatively isolated areas that are not subject to frequent reintroductions (e.g. islands), but which have realistic levels of rabies surveillance (i.e. case-detection probabilities < 10%), mass dog vaccination is more effective at controlling rabies (and is no more costly) than vaccinating only in response to detected outbreaks. These models further indicate that surveillance measures need to be able to detect at least 5% of rabies cases in order to be confident that rabies has been eliminated under the current guidelines for declaration of freedom (Townsend *et al*. 2012).

These models were developed initially for populations without repeat introductions but, in reality, high levels of legal and illegal dog movements remain a concern for maintaining rabies-free status in areas where the disease has been eliminated. Informal movement of dogs through road and water-based transport can represent a considerable challenge in many areas and, whilst cross-boundary coordination and effective rabies control over large geographic regions should mitigate the risk of new introductions, effective surveillance will still be needed in order to detect and respond to new incursions. Maintaining surveillance levels is also important so as to detect outbreaks that may be triggered by rare spill-over transmission from other reservoir hosts, such as bats, and to contain outbreaks that might otherwise have the potential to establish new epidemic cycles.

With recognition of the critical importance of strengthening rabies surveillance, much effort is therefore being directed at improvements in detection, reporting and laboratory diagnosis of rabies. Examples include the application of mobile phone technologies to enhance reporting of human rabies exposures at bite treatment centres in Tanzania (Mtema 2013). Mobile phone systems also allow for rapid communication between human and animal health sectors to ensure follow up of animal cases; reminder texts to be sent to patients to complete the full course of PEP; and improved distribution of vaccine stocks to avoid the vaccine shortages that occur frequently in many of the more remote parts of Africa (Mtema 2013).

The advent of new diagnostic techniques is also helping to overcome some of the logistic, technical and cultural barriers associated with submitting human and animal samples for centralised laboratory diagnosis using conventional fluorescence techniques. These include (1) new diagnostic tests that use light microscopy rather than fluorescence microscopy and can therefore be carried out at local facilities with very high specificity and sensitivity (Dürr *et al*. 2008; Lembo *et al*. 2006); (2) field-based lateral flow devices, that may provide a useful tool for generating surveillance data (Markotter *et al*. 2009) and for empowering and incentivising field workers to engage with rabies surveillance (Halliday *et al*. 2012); (3) new techniques for *intra vitam* diagnosis of human rabies cases (Dacheux *et al*. 2008; Fooks *et al*. 2009); and (4) culturally-acceptable methods for post-mortem sampling of brain material for human rabies diagnosis (Mallewa *et al*. 2007).

Although these techniques are improving capacity for laboratory-confirmed diagnosis in Africa, attention also needs to be given to improving the reporting of clinical cases, which provides the entry point for building an effective rabies surveillance system. Whilst laboratory diagnosis provides robust confirmation of rabies and is important for effective administration of PEP, clinical case reporting also needs strengthening as the central pillar of rabies surveillance. For example, surveillance in the smallpox eradication programme was underpinned by clinical case reporting from health centres (Henderson & Klepac 2013) and during the rinderpest eradication campaigns in Africa, participatory surveillance based on the engagement of local communities was the primary surveillance tool in the final stages of eradication (Roeder, Mariner & Kock 2012).

Given the distinctive nature of dog rabies, recognition amongst local communities in Africa can be high (e.g. 74% of cases reported as suspect rabid animals in rural Tanzania were confirmed positive on laboratory diagnosis; Lembo *et al*. 2008). Whilst clinical surveillance is clearly neither 100% specific nor sensitive, these cases can contribute valuable epidemiological data for the comparative analysis of trends, initiating follow-up investigations and, most critically, providing a stimulus for disease control measures. Indeed, the lack of feedback or beneficial response to mitigate disease problems is arguably the greatest barrier to the reporting of zoonoses, particularly within resource-poor systems, and this chronic lack of response (or capacity to respond) is disempowering and demotivating at the grassroots level for healthcare and veterinary workers alike (Halliday *et al*. 2012). Therefore, whilst there is no doubt that laboratory diagnostic capacity needs strengthening throughout most of Africa, the lack of capacity should not act as a deterrent to initiating rabies control measures. Rather, effective rabies control measures should be integrated within responsive surveillance systems and should provide motivation to field staff to improve case reporting and submission of samples.

### Economic analyses

Whilst confidence is growing as to the epidemiological feasibility of canine rabies control and elimination through mass dog vaccination, a key question relates to the cost and sustainability of control measures, particularly in low-income countries. In Tanzania, mass vaccination campaigns have led to dramatic declines in demand for PEP (Cleaveland *et al*. 2003) and the concomitant savings to the public health sector generate a potential mechanism for sustaining dog vaccination campaigns. Cost-effectiveness analysis, incorporating deterministic models of dog-to-human transmission, indicate that strategies involving dog vaccination and PEP are likely, over the longer term, to be more cost effective for preventing human rabies deaths than human PEP alone (Bögel & Meslin 1990; Zinsstag *et al*. 2009).

However, the relationship between dog rabies incidence and human PEP demand, which is a critical determinant of cost-effectiveness, is likely to vary considerably across different socioeconomic settings. In northern Tanzania, for example, the incidence of bite injuries reported at health clinics declines rapidly as the incidence of rabies falls (Cleaveland *et al*. 2003) and can decline to zero in areas where canine rabies has been eliminated (Lembo *et al*. 2010). This suggests that people in these communities seek PEP only when they recognise the risk of rabies – they would not seek treatment for bite injuries from non-rabies suspect animals, a situation which presumably reflects both a high level of rabies recognition and the high private costs associated with PEP in comparison with household incomes in Africa (Knobel *et al*. 2005). In contrast, in many higher-income settings or where rabies occurs more sporadically, the scale of PEP use may be driven both by health-seeking behaviour of more affluent and knowledgeable members of the community as well as clinician decision making. In these situations, pressures and uncertainties faced by clinicians regarding the genuine need for PEP means that PEP use can remain very high even when the incidence of animal rabies cases and risk of exposure to rabies is extremely low (Lardon *et al*. 2010), with important implications for the cost-effectiveness of human rabies prevention through rabies control in the animal reservoir.

Research findings have also contributed important information for practical field operations, including fundamental questions relating to dog population ecology and ownership patterns, which are central to the design of rabies control measures.

### Dog ecology studies

An enduring misperception relating to dog ecology in Africa is the widely-held, but erroneous, impression that a large proportion of dogs are ownerless or ‘stray’ dogs that are not accessible for vaccination. This has had major implications for rabies control across Africa, with policy makers being reluctant to invest in dog vaccination campaigns and resources rather being directed toward ineffective strategies, such as culling (Figure 1).

A key finding from dog ecology studies is that, although most dogs in Africa are free-roaming, the number of ownerless dogs or those inaccessible for vaccination remains very low. For example, only 1%, 8% and 11% of dogs respectively were unowned in three study sites in N’Djamena (Kayali *et al*. 2003). In Zimbabwe, all dogs on communal lands were owned (Butler & Bingham 2000) and in Tanzania, < 1% of dogs were unowned in an urban site that was specifically targeted for dog ecology studies on the basis of reports of a large population of ‘stray’ dogs (Gsell *et al*. 2012). Although precise estimates of ownerless dogs are difficult to obtain, a robust conclusion remains that the level of dog ownership and dog accessibility is sufficiently high in all these communities to be able to control dog rabies through mass dog vaccination of owned dogs.

Estimating the size of dog populations has been considered a key operational requirement for mass dog vaccination campaigns. However, although several approaches to determining the dog population size have been described (summarised in the Canine Rabies Blueprint [Global Alliance for Rabies Control 2013]), difficulties still remain.

For owned dogs, knowledge of the human:dog ratio (HDR) combined with human population data can provide useful preliminary estimates. Dog ecology studies from across Africa indicate relatively consistent HDR values in rural settings (Davlin & Vonville 2012; Knobel *et al*. 2008), but the HDR can vary widely, particularly in urban settings. Furthermore, human population figures are generally obtained from census data, which may only be collected every 10 years, and projections from these data are usually generated from average population growth rates. However, with rapid changes in human demographics, including high rates of urbanisation in Africa (UN Habitat 2008), population growth rates at a local level can vary widely, leading to uncertainties in projected population sizes and, hence, dog population estimates. Therefore, whilst an initial crude estimate of the dog population size can be generated using the projected human population sizes and ‘average’ or ‘typical’ HDRs for planning of first campaigns, these figures should be refined progressively as campaigns are implemented.

An important point in designing household dog population surveys is that estimates of HDR need to explicitly include pups, because pups are not always considered as ‘dogs’ in answer to the question about the number of dogs in a household. It is also important to note that young pups (less than 3 months of age) need to be included for rabies vaccination during annual campaigns (a point that is often not well recognised by either the owners of puppies or veterinary officers). Determining vaccination coverage levels for puppies separately from adults can be useful in order to identify whether low coverage in pups is a problem that needs to be addressed.

Despite uncertainties in most approaches to estimating dog population sizes, it is clear that extremely precise data may not be critical at the initial stages of implementing national strategies and more precise data can be generated as campaigns are rolled out. For procurement purposes, estimates should over-estimate rather than underestimate dog population sizes; provision of excess vaccines at the start of a rabies control programme is generally not problematic, provided the shelf life allows for vaccine to be used in successive campaigns.

In addition to planning campaigns, knowledge of the dog population size is also important in order to determine vaccination coverage, a critical parameter for monitoring vaccination campaigns. During campaigns, vaccination coverage is often determined from the number of dogs vaccinated during a campaign (or vaccine doses used) divided by the estimated dog population size, but given the uncertainties in dog population sizes, these figures are also likely to be unreliable. It should also be noted that, during recurrent campaigns, using vaccine doses to estimate coverage would only provide a conservative estimate, as dogs vaccinated in the previous year(s) and not during the current campaign would not be included in coverage estimates. However, if using vaccines with 2–3 years’ duration of immunity (as is the case for most commercial vaccines), some of these dogs may still be protected against rabies and contributing to population immunity. Because of the critical importance of monitoring vaccination coverage during national control programmes, alternative methods should be adopted to generate more precise values at the community level. Household surveys generate accurate and useful data and it is feasible to include them in vaccination campaigns that adopt ‘house-to-house’ strategies. However, logistic and resource constraints may preclude sampling other communities and, as the ‘even-ness’ of coverage is likely to be an important measure (Townsend *et al*. 2013), greater emphasis may need to be given to developing relatively cheap and simple population- and community-based survey tools that could be applied more broadly in all vaccinated communities. Other forms of post-vaccination survey include mark-resight surveys, which capture coverage in free-roaming dogs (e.g. Kaare *et al*. 2009; Kayali *et al*. 2003; Matter *et al*. 2000) and could be encouraged as standard methodology for campaigns which are conducted using centralised vaccination stations and in areas with a high proportion of free-roaming dogs. These methods have, in addition, been used to estimate the size of the ownerless dog population (e.g. Gsell *et al*. 2012).

### International partnerships

With strong interdisciplinary partnerships now established, involving the Partners for Rabies Prevention, the tripartite partnership of the World Health Organization (WHO), the Food and Agriculture Organization of the United Nations (FAO) and the World Organisation for Animal Health (OIE), as well as other international health and animal welfare agencies, there is a growing international momentum toward canine rabies elimination (Lembo *et al*. 2011). Rabies has been included within the WHO Roadmap for Neglected Tropical Diseases (WHO 2012), with targets set for regional elimination of canine rabies in Latin America by 2015 and South-East Asia by 2020. To support the development of national rabies control plans, a step-wise strategy has been developed (FAO 2013), identifying the key activities and capacities that need to be established at different stages of disease control and elimination. This framework is further supported by practical guidelines (the ‘Canine Rabies Blueprint’) in order to support field operations (Lembo 2012).

## Conclusion

In summary, there are clearly strong grounds for optimism that canine rabies can be controlled and eliminated in Africa. Multiple strands of evidence from empirical and theoretical research studies generate confidence as to the epidemiological feasibility of canine rabies elimination, with the principal focus of activities directed toward mass dog vaccination. In addition, findings from operational research studies, in tandem with strong international partnerships, provide practical guidance and support needed for the design and implementation of effective national and regional elimination programmes.
